# Viral carcinogenesis: virus implicated in cancer

**DOI:** 10.1186/1753-6561-7-S2-K11

**Published:** 2013-04-04

**Authors:** Luisa Lina Villa

**Affiliations:** 1 Dept of Radiology and Basic Oncology, School of Medicine, USP, São Paulo, Brazil; 2 HPV Institute, Santa Casa de São Paulo, São Paulo, Brazil

## 

The first consistent observations that viruses could be associated with some types of cancer where made almost a century ago. A great deal of effort was involved in unraveling the molecular mechanism underlying carcinogenesis implicated to animal and human viruses. As a result of these studies, a strong link between some viral agents and several human cancers has been established. Some viruses as the Epstein-Barr virus (EBV), hepatitis B virus (HBV), hepatitis C virus (HCV), human T-cell lymphotropic virus type I (HTLV-I), immunodeficienciy virus type I (HIV-1) and several human papillomavirus types (including types 16, 18, 31, 33, 35, 39, 45, 51, 52, 56, 58, 59 and 66) have been classified as group 1 carcinogens by the International Agency for Research in Cancer (IARC). Infection by these viruses constitutes a heavy burden for human populations as it accounts for almost 15% of all human malignancies. Furthermore, many other viral agents have been classified as probably (group 2A carcinogens) or possibly (group 2B carcinogens) carcinogenic to humans and others have been occasionally found in human tumors suggesting that this figure is just an underestimation of viral involvement in human cancer etiology. Nevertheless, viral infection appears as one of the main cancer risk factors that could be prevented. Prevention and control of infection by these agents could dramatically reduce the incidence of some prevalent cancers and, consequently, have a great impact on public health.

## Competing interests

There are no competing interests in this presentation.

